# Living and learning with a blind mind’s eye: college students with aphantasia

**DOI:** 10.3389/fpsyg.2025.1615860

**Published:** 2025-10-30

**Authors:** Jenel T. Cavazos, Hannah M. Baskin, Taylor M. Mashigian, M. Anthony Cavazos

**Affiliations:** ^1^Department of Psychology, University of Oklahoma, Norman, OK, United States; ^2^Department of Communication, University of Oklahoma, Norman, OK, United States

**Keywords:** aphantasia, hyperphantasia, student success, visual imagery, college students

## Abstract

**Introduction:**

Aphantasia, the inability to voluntarily generate visual mental imagery, affects approximately 2–5% of the population. The current study investigated how college students with aphantasia navigate academic environments despite lacking this cognitive ability, which is traditionally considered fundamental to learning.

**Method:**

Study 1 quantitatively examined relationships between visual imagery ability and academic variables among 450 college students, while Study 2 qualitatively explored the experiences of 14 aphantasic college students through semi-structured interviews.

**Results:**

While hyperphantasic students demonstrated significantly higher episodic memory, future thinking ability, and greater use of certain study behaviors (practice testing and explanation generation), no significant differences emerged in deep, strategic, or surface learning approaches. Qualitative analyses revealed four major compensatory mechanisms: (1) extensive externalization through list-making and organizational systems; (2) systematic verbal processing strategies; (3) anchoring new information to familiar references; and (4) multi-modal approaches to visual-heavy content.

**Conclusion:**

These findings demonstrate that aphantasic students systematically externalize cognitive processes that others typically internalize through visualization. Despite lacking mental imagery, these compensatory strategies enable aphantasics to perform academically as well as their peers. This research highlights the brain’s remarkable adaptability and suggests approaches for creating more inclusive learning environments that accommodate diverse cognitive profiles.

## Introduction

Mental imagery, the ability to create and manipulate mental representations of objects or scenes not physically present, plays a crucial role in cognitive processes such as memory, learning, and problem-solving (Dawes et al., 2020; Kay et al., 2021; Kosslyn et al., 2006). However, approximately 2%–5% of the population experiences a condition known as aphantasia, which is characterized by the inability to voluntarily generate visual mental images (Zeman, 2024). Though only recently defined, this phenomenon has roots in psychological and neurological inquiry dating back to the late 19th century. Sir Francis Galton was among the first to document significant variability in mental imagery abilities, noting that “scientific men as a class have feeble powers of visual representation,” challenging the then-prevailing assumption that mental imagery was universal. Despite these early observations, the systematic study of individuals without mental imagery remained largely unexplored for over a century, partly due to limitations in scientific methodologies for studying subjective experiences. The formal recognition of aphantasia as a distinct cognitive condition emerged only recently through Zeman et al.’s (2015) groundbreaking work on individuals who experience the lifelong absence of mental imagery. This work catalyzed a rapid expansion of research into the diversity of mental imagery experiences and their implications for cognition processes such as learning and memory.

Research has provided compelling physiological evidence to support the existence of aphantasia. For example, Kay et al. (2021) demonstrated that individuals with typical visual imagery ability show a predictable pupillary light response when imagining bright objects, while aphantasic individuals do not. Keogh and Pearson (2018) found that individuals with self-reported aphantasia demonstrate no priming effect on binocular rivalry tasks, which supports the conclusion that aphantasics experience the objective absence of sensory imagery. Wicken et al. (2021) expanded our understanding of aphantasia by documenting reduced physiological responses (specifically, diminished galvanic skin response) in aphantasics during imagery-dependent emotional tasks, such as imagining fear-inducing stories. Recently, Dupont et al. (2024) used transcranial magnetic stimulation to reveal that aphantasic individuals lack the increases in corticospinal excitability during both kinesthetic and visual imagery that are characteristic of those with typical imagery abilities. These findings align with cognitive neuroscience research suggesting that mental imagery involves accessing perceptual information from memory in a way similar to “seeing with the mind’s eye” (Kosslyn et al., 2001), with aphantasia representing a disruption in this process.

Assessment of aphantasia primarily relies on self-reported measures of visualization ability. The most commonly used measure is the Vividness of Visual Imagery Questionnaire (VVIQ; Marks, 1973), which has demonstrated high reliability in multiple studies (Campos, 2011; Larner et al., 2024; Milton et al., 2021). The VVIQ asks participants to visualize a series of specific scenes and objects and rate the vividness of their mental images on a Likert-type scale. VVIQ scores range between 16 and 80. The operationalization of aphantasia varies across studies, with some using only complete absence (VVIQ = 16; Fulford et al., 2018; Zeman et al., 2015), while others use broader ranges such as 16–23 (Milton et al., 2021; Zeman et al., 2020) or 16–25 (Bainbridge et al., 2021). Zeman et al. (2020) distinguished between “extreme aphantasia” (score of 16) and “moderate aphantasia” (scores 17–23), collectively defining aphantasia as VVIQ scores < 24. This places aphantasia at one end of a broader spectrum of visualization abilities that extends to hyperphantasia (extremely vivid imagery, typically defined as scores 75–80) at the opposite end.

### Theoretical frameworks for understanding imagery and learning

One important implication of this research is the impact of imagery on learning and memory. Multiple theoretical perspectives offer competing views on the role of visual imagery in learning and cognition. Dual Coding Theory (Paivio, 1991) proposes that cognition involves two separate but interconnected systems: a verbal system specialized for linguistic information and a nonverbal (imagery) system that is specialized for processing nonverbal objects and events. According to this theory, information encoded through both systems creates multiple retrieval pathways, enhancing memory and learning. Research has supported this conclusion by demonstrating that visual imagery enhances learning outcomes across various educational contexts and age groups, including math and writing abilities in young children, reading comprehension in middle-school students, and comprehension of scientific texts in college students (Guarnera et al., 2019; Jenkins, 2009; Leopold and Mayer, 2015). Studies have further demonstrated a relationship between vivid mental imagery and improved semantic, episodic, and autobiographical memory (D’Angiulli et al., 2013) as well as prospective memory (Scullin et al., 2017). According to Dual Coding Theory, imagery functions as a powerful cognitive system that can reduce mental load and improve retention of information, with meaningful learning often requiring cognitive processes that link visual and verbal information (Mayer and Moreno, 2003).

However, alternative theoretical perspectives suggest that visual imagery may not be central to all learning processes. For example, working memory models (Baddeley, 2000) propose multiple component systems that can operate independently, suggesting that verbal and spatial processing can compensate for one another. Similarly, embodied cognition theories emphasize that cognition extends beyond mental imagery to include motor, kinesthetic, and other sensory-based representations (Barsalou, 2008). These frameworks suggest that successful learning may not require visual imagery *per se*, but rather effective encoding and retrieval mechanisms that can be achieved through multiple cognitive pathways.

The existence of aphantasia provides a unique opportunity to examine these competing theoretical predictions. If visual imagery is fundamental to learning (as strict interpretations of Dual Coding Theory might suggest), aphantasic students should show marked deficits or rely predominantly on verbal strategies. However, if multiple cognitive pathways can support learning (as working memory and embodied cognition theories suggest), aphantasic students might develop diverse compensatory mechanisms that achieve similar outcomes through alternative routes.

Despite the absence of voluntary visual mental imagery, individuals with aphantasia do not exhibit deficits in standardized measures of cognitive ability such as IQ (Milton et al., 2021; Pounder et al., 2022). Research has consistently found no significant differences in general neuropsychological functioning between aphantasic and non-aphantasic individuals (Pounder et al., 2022), and aphantasic students appear to achieve comparable academic outcomes to their peers despite lacking this cognitive ability. This pattern–preserved cognitive functioning despite lacking an ability often considered fundamental–suggests that aphantasic individuals employ alternative cognitive strategies to compensate for their lack of visual imagery. Research suggests that the compensation (or adaptation) process used by aphantasics appears to be unconscious, virtually instant, and capable of rendering their performance on par with those who experience “normal” visual imagery. For example, spatial imagery abilities remain largely intact despite absent visual object imagery, with aphantasics performing as well as controls on spatial imagery questionnaires and mental rotation tasks (Dawes et al., 2022; Keogh and Pearson, 2018; Azañón et al., 2025). Similarly, Keogh et al. (2021) observed that aphantasics were able to complete visual working memory tasks (such as asking participants to recall the colors and positions of squares, dots, and lines after a short delay) just as well as non-aphantasics. The authors suggested that aphantasics have some form of “unconscious” working memory and that there must be an “underlying difference in the neural mechanisms that aphantasics use to hold visual information in mind” (p. 250). Arcangeli (2023) observed that aphantasia extends beyond visual impairments to affect multisensory experiences, suggesting that compensatory mechanisms likely operate across multiple cognitive domains. These findings raise important questions about the nature of cognitive processes in the absence of voluntary mental imagery.

One potential domain where individual differences in visual imagery might manifest is in students’ approaches to studying and encoding information. Successful learning in college environments requires effective encoding strategies, and research has identified three primary approaches to studying: deep, strategic, and surface (Biggs, 1987; Graham et al., 1984). Deep approaches to studying involve seeking meaning and understanding, often through techniques such as creating personal examples or concept mapping (Asikainen et al., 2020). Strategic approaches focus on achieving high grades through time management and efficient study methods, such as prioritizing work based on point values and analyzing past exam questions to predict what will be tested (Brown and Murdolo, 2016). Surface approaches emphasize techniques for rote memorization without deep engagement (such as highlighting notes or reviewing flashcards; Lindblom-Ylänne et al., 2019). The effectiveness of these approaches has been linked to various academic outcomes. For example, the use of deep and strategic approaches is associated with higher GPAs, reduced academic exhaustion, and improved problem-solving skills (Kusurkar et al., 2013; Ward, 2011). Conversely, surface approaches are correlated with lower GPAs and higher rates of academic burnout (Asikainen et al., 2020; Richardson et al., 2012).

These established learning approaches, however, typically assume students have intact mental imagery abilities. Research specifically examining visualization ability as an individual difference variable in academic learning contexts has been more limited compared to experimental studies that use visualization as an independent variable, manipulating instructions to visualize while presenting a learning task and measuring the subsequent effects on recall (Abel et al., 2024; McFarland and Glisky, 2011). While educational psychology has established extensive research on individual differences in learning preferences, fewer studies have specifically examined how natural variations in visualization ability – particularly extreme variations like aphantasia – might affect academic learning processes and compensatory strategies. The question of how aphantasic students navigate different study approaches while achieving comparable academic outcomes provides an opportunity to test these competing theoretical predictions and understand the flexibility of human cognition.

This line of research reveals a gap in our understanding of how aphantasic students effectively encode and retrieve information. For educators and researchers, understanding these alternative cognitive strategies could provide valuable insights into diverse learning processes and inform more inclusive teaching methods that benefit all students, regardless of their imagery abilities.

To explore the relationship between the spectrum of visual imagery and learning, we designed a two-part mixed-methods study:

1. A quantitative analysis examining the associations between visual imagery ability and academic variables including study approaches (deep, strategic, and surface), learning preferences, memory and metacognitive awareness, and academic self-efficacy. This phase allowed us to identify patterns across the imagery spectrum while controlling for potential confounding variables.
2. A qualitative analysis of semi-structured interviews with aphantasic college students to understand their lived educational experiences, identify compensatory strategies, and explore how the absence of visual imagery influences their learning processes, study techniques, and academic interactions.

This mixed-methods design allows us to (1) measure broad patterns in learning approaches, and (2) deeply explore the experiences of aphantasic students. We anticipate that aphantasic students have adapted unique approaches to learning that could inform the development of more inclusive teaching methods and personalized study approaches that accommodate diverse cognitive profiles. The research questions guiding this project are:

1. How do varying levels of visual imagery ability correlate with preferred study strategies and academic performance variables?
2. What compensatory mechanisms do aphantasic students develop to support their learning?
3. How might understanding these differences inform educational practices in the broader community?

By addressing these questions, we aim to contribute to both the theoretical understanding of aphantasia and practical applications for supporting diverse learners in higher education.

## Study 1

Study 1 was designed to explore the relationship between study habits and visualization ability. In contrast to previous research on visualization and learning, which often present visualization as an independent variable, our study is interested in individual differences in visual imagery ability as it relates to academic strategies and outcomes. Based on established research showing that mental imagery enhances learning across various contexts (Guarnera et al., 2019; Jenkins, 2009; Leopold and Mayer, 2015), we hypothesized that students with stronger visual imagery abilities would demonstrate a tendency to use deep study approaches, which often rely on creating meaningful connections and visual-conceptual links to existing knowledge structures. We also anticipated they would show a preference for instructors who promote a comprehensive understanding of material, enhanced episodic and autobiographical memory performance, and greater metacognitive awareness and academic self-efficacy.

In contrast, we proposed that aphantasic students would report more challenges with episodic and autobiographical memory tasks. We initially hypothesized that aphantasic students might rely on surface-level approaches to learning, reasoning that these students would need alternative strategies when deeper approaches that require visual-conceptual anchoring proved challenging. However, we acknowledge this prediction was theoretically problematic, as surface approaches often emphasize visual features and rote memorization techniques that may themselves depend on visual processing. Given the limited research on learning strategies in aphantasia, our predictions about study approaches were necessarily exploratory. By examining correlations across the full spectrum of imagery ability, our goal was to identify specific patterns that could inform more inclusive pedagogical practices and reveal the compensatory mechanisms that allow aphantasic students to succeed academically despite lacking visual mental imagery.

## Method

### Participants

A total of 669 students enrolled in an introductory psychology course at a large Midwestern university participated in this study in return for credit toward a class research requirement. The majority of participants were 18–19 years old (*n* = 384, 86%), and 78% were first-year students (*n* = 351). Of the total sample, 292 participants (65%) identified as female, 154 (34%) identified as male, and 7 (1.6%) identified as non-binary. The racial/ethnic composition of the sample was 77% White (*n* = 345), 10% Latinx (*n* = 45), 9% American Indian or Alaska Native (*n* = 40), 8.7% Asian (*n* = 39), 7.6% Black (*n* = 34), and 0.4% Native Hawaiian or Pacific Islander (*n* = 2).

#### Materials

##### Approaches and study skills inventory for students

To investigate students’ study habits, participants were asked to complete the Approaches and Study Skills Inventory for Students test (ASSIST). The ASSIST scale was designed to measure students’ relative use of three different approaches to studying: deep, strategic, and surface (Entwistle and Tait, 2013). The ASSIST consists of 26 statements, 18 related to study habits, and eight related to preferences for the course and teaching methods. An example statement related to the deep study approach reads: “When I read an article or book, I try to find out for myself exactly what the author means.” An example statement related to the strategic approach reads: “I think I’m quite systematic and organized when it comes to revising for exams.” An example statement related to the surface study approach reads: “Much of what I’m studying makes little sense: it’s like unrelated bits and pieces.” Each of the study approaches subscales demonstrated adequate reliability; α = 0.67 (deep), 0.81 (strategic), and 0.73 (surface).

The Preferences for Different Types of Courses and Teaching Scale is an 8-question extension of the ASSIST that asks students to indicate a preference for teaching methods focused on transmitting information or those that promote a deep understanding of material. Participants are instructed to indicate their agreement with each statement on a 1 (disagree) to 5 (agree) Likert-type scale. An example statement from the transmitting information subscale is, “Lecturers who tell us exactly what to put down in our notes.” An example statement from the deep understanding subscale is, “Lecturers who encourage us to think for ourselves and show us how they, themselves, think.” Both subscales demonstrated adequate reliability; α = 0.66 (transmit information) and α = 0.68 (deep understanding).

##### Vividness of visual imagery questionnaire

To investigate mental imagery ability, participants were asked to complete the Vividness of Visual Imagery Quiz (VVIQ). The VVIQ is used to measure individual differences in the ability to create vivid mental images (Eton et al., 1998). It is made up of four scenarios, each containing four statements, for a total of sixteen statements. Participants were instructed to read carefully and attempt to form a mental picture of the scenarios given to them. An example prompt from the VVIQ asks the participant to imagine the specific details of an absent friend or family member’s face, body, and other physical features. Participants reported the vividness of their visual images on a Likert-type scale ranging from 1 (No image at all. I only “know” that I am thinking of the object) to 5 (Perfectly realistic, as vivid as the real thing). The VVIQ demonstrated excellent reliability; α = 0.91.

##### Object and spatial imagery questionnaire (OSIQ)

To further understand internal representations of visual imagery, participants were asked to complete the Object and Spatial Imagery Questionnaire (OSIQ) evaluates individual preferences for object and spatial imagery, using a 5-point Likert scale (1 = Strongly Disagree, 5 = Strongly Agree). Object imagery refers to the ability to form detailed mental images, as illustrated by phrases like, “My mental images are very vivid and detailed.” In contrast, spatial imagery applies to the ability to manipulate spatial relationships mentally, with statements such as, “I can easily imagine how a room would look from a different angle.” Higher scores on each subscale indicate a stronger particular imagery style; α = 0.89.

##### Survey of autobiographical memory

The Survey of Autobiographical Memory (SAM) is a 26-item questionnaire that assesses individual differences in the self-reported effectiveness of autobiographical, semantic, spatial, and episodic memory (Picco et al., 2020). Participants used a Likert-type scale ranging from 1 (strongly disagree) to 5 (strongly agree) to rate their level of agreement with each statement. An episodic memory statement is: “After I have met someone once, I easily remember his or her name.” A semantic memory example is: “I can learn and repeat facts easily, even if I don’t remember where I learned them.” A spatial memory example is: “I have a hard time judging the distance (e.g., in meters or kilometers) between familiar landmarks.” An example future memory statement is: “When I imagine an event in the future, the event generates vivid mental images that are specific in time and place.” Each memory subscale demonstrated sufficient reliability: episodic memory α = 0.79; semantic memory α = 0.60; spatial memory α = 0.78, and future memory α = 0.86.

##### Motivated strategies for learning questionnaire

The Motivated Strategies for Learning Questionnaire (MSLQ; Pintrich and de Groot, 1990) is a 44-item scale that captures motivational beliefs and self-regulated learning. It includes five subscales (three assessing motivational beliefs and two assessing self-regulated learning), each of which are assessed using a Likert-type scale ranging from 1 (not at all true of me) to 7 (very true of me). Participants were instructed to think about a specific course (in this case, their psychology class) when responding to the items.

The first motivational beliefs subscale consists of nine items designed to measure self-efficacy. An example item from this subscale is, “I’m certain I can understand the ideas taught in this course.” The scale showed adequate reliability; α = 0.91. The second motivational beliefs subscale consists of nine items that measure the intrinsic value of the information learned in the course. An example item from this subscale is, “I think I will be able to use what I learn in this class in other classes.” The scale showed adequate reliability; α = 0.86. The last motivational beliefs subscale is a four-item measure of test anxiety. An example item from this subscale is, “I have an uneasy, upset feeling when I take a test.” The scale showed adequate reliability; α = 0.89.

The first self-regulated learning subscale consists of 13 items that measure cognitive strategy use. An example item from this subscale is, “When studying, I copy my notes over to help me understand the material.” The scale showed adequate reliability; α = 0.79. The second self-regulated learning subscale includes nine items that are designed to capture self-regulation. An example item from this subscale is, “Even when study materials are dull and uninteresting, I keep working until I finish.” The scale showed adequate reliability; α = 0.71.

##### Study behaviors checklist

The Study Behaviors Checklist (SBC) is a 34-item self-report questionnaire that assesses a student’s organizational and study behaviors (Gurung et al., 2010). Students rated their level of agreement on a five-point Likert scale ranging from 1 (not like me at all) to 5 (exactly like me). Previous research using the SBC has used the items both as individual predictors (Gurung et al., 2010) and grouped as behavior types (Bartoszewski and Gurung, 2015). To determine the most appropriate variable structure, principal axis factoring with Promax rotation was conducted (Field, 2013). The analysis yielded four study behavior categories explaining a total of 51.125% of the variance for the entire set of variables.

The first study behavior category was labeled Organization Behaviors. This category includes eight items with statements such as, “I divide material into smaller, manageable, and logical sections,” and “I actively modify my studying for different exam formats.” This factor explained 29.139% of the variance and showed adequate reliability; α = 0.80.

The second study behavior category was labeled Note Taking Behaviors. This category includes five items with statements related to note-taking, such as, “My notes are well-organized,” and “I take notes on what I am reading.” This factor explained 9.282% of the variance and showed adequate reliability; α = 0.83.

The third study behavior category was labeled Explanation Behaviors. This category included five items with statements such as, “I generate my own examples about the material,” and “I am able to explain a problem or phenomenon using the material.” This factor explained 6.568% of the variance and showed adequate reliability; α = 0.72.

The fourth study behavior category was labeled Testing Behaviors. This category included four items with statements such as, “I use practice exams to study,” and “I test myself without any notes.” This factor explained 6.136% of the variance and showed adequate reliability; α = 0.72.

##### Operational definitions of aphantasia and hyperphantasia

Following established precedent, we operationally defined aphantasia as VVIQ scores from 16 to 24, consistent with Zeman et al.’s (2020) recommendation of <24. This range encompasses both extreme aphantasia (complete absence, score = 16) and moderate aphantasia (scores 17–23), capturing individuals with severely deficient to completely absent visual imagery. Hyperphantasia was defined as VVIQ scores from 75 to 80, following Milton et al. (2021) and Zeman et al. (2020).

The asymmetric ranges reflect the conceptual definitions of these conditions: hyperphantasia represents imagery that is “perfectly clear and as vivid as normal vision,” requiring near-perfect ratings across VVIQ items and creating a narrow ceiling effect. In contrast, aphantasia encompasses a broader range from completely absent to severely deficient imagery, allowing for greater variability at the lower end of the scale. This asymmetry arises from the conditions themselves rather than statistical properties of the VVIQ distribution.

#### Procedure

Participants were recruited via the psychology department experiment management system and were then redirected to Qualtrics, an online survey system, to indicate informed consent and to complete the survey. After the survey, participants were debriefed, and the session was concluded. A research assistant then reviewed each participant’s responses to four embedded attention check items and granted credit within the experiment management system accordingly. Participants who failed more than one of the embedded attention checks were not given credit for participation, and their data were excluded from analyses (*n* = 219). This left a final sample of 450 participants, with 21 of those participants scoring below a 24 on the VVIQ, indicating these participants likely have Aphantasia, and 36 of those participants scoring above a 75 on the VVIQ, indicated they likely have Hyperphantasia.

### Results

To examine our hypotheses, a multivariate multiple regression (MMR) was conducted with scores on the VVIQ predicting scores on our primary outcome measures. Wilk’s lambda was used to test the omnibus hypothesis that VVIQ total scores significantly predicted the outcome variables. This test was statistically significant, *F*(16, 389) = 6.59, *p* < .001, R^2^ = 0.213. Consequently, we explored the relationship between the VVIQ and each individual variable (see Table 1 for a summary of the results). Given the multiple univariate regression tests conducted (16 outcome variables), we applied the Benjamini-Hochberg False Discovery Rate (FDR) correction to control for inflated Type I error rates in the regression analyses only (see Table 2 for summary of the results). Group comparison ANOVAs were analyzed separately without correction as they represent distinct analytical questions.

**TABLE 1:** Outcome variables from MMR analysis with VVIQ scores as predictors.

Outcome variable	*β*	*F*(1, 405)	*p*	R^2^
ASSIST strategic subscale	0.056	7.671	0.006	0.019
ASSIST deep subscale	0.043	4.708	0.031	0.012
ASSIST surface subscale	−0.014	0.472	0.492	0.001
ASSIST transmit information subscale	0.016	2.842	0.093	0.007
ASSIST support understanding subscale	0.019	2.023	0.156	0.005
SAM prospective memory subscale	0.028	63.310	<0.001	0.135
SAM episodic memory subscale	0.018	36.058	<0.001	0.082
SAM semantic memory subscale	0.007	5.597	0.018	0.014
SAM spatial memory subscale	0.006	2.716	0.100	0.007
MSLQ self-efficacy subscale	0.013	10.239	0.001	0.025
MSLQ intrinsic value subscale	0.007	3.099	0.079	0.008
MSLQ test anxiety subscale	−0.001	0.007	0.933	0.000
SBC organization behaviors factor	0.015	21.526	<0.001	0.051
SBC explanation behaviors factor	0.014	18.742	<0.001	0.044
SBC testing behaviors factor	0.015	15.378	<0.001	0.037
SBC note taking behaviors factor	0.006	2.157	0.143	0.005

**TABLE 2:** Benjamini-Hochberg FDR correction summary.

Variable	Original *p*	FDR-adjusted *p*
Autobiographical memory	<0.001	<0.001
Episodic memory	<0.001	<0.001
Organization behaviors	<0.001	<0.001
Explanation behaviors	<0.001	<0.001
Testing behaviors	<0.001	0.001
Self-efficacy	0.001	0.008
ASSIST strategic	0.006	0.024
Semantic memory	0.018	0.048
ASSIST deep	0.031	0.083
Explanation behaviors (group)	0.025	0.067

6 additional variables with *p* > 0.079 remained non-significant after FDR correction.

To better understand the relationship between individual differences in visualization ability and our outcome variables, we created groups composed of the extreme ends of the visualization spectrum. The hyperphantasic group included those who scored between 75 and 80 (out of a possible high score of 80) on the VVIQ, while the aphantasic group included those who scored between 16 and 24, encompassing both extreme (16) and moderate (17–23) aphantasia as defined by Zeman et al. (2020). A series of one-way ANOVAs were conducted to examine group differences across outcome variables. Means and standard deviations for the statistically significant Aphantasia and Hyperphantasia group comparisons can be found in Table 3.

**TABLE 3:** Means and standard deviations for statistically significant group comparisons.

Measure	Aphantasia M (SD)	Hyperphantasia M (SD)
SAM - episodic memory	3.11 (0.97)	3.96 (0.75)
SAM - future thinking	2.15 (1.06)	4.19 (0.71)
MLSQ - intrinsic motivation	5.53 (0.90)	6.15 (0.72)
SBC - overall score	3.18 (0.53)	3.83 (0.69)
OSIQ - object imagery	2.31 (0.64)	4.19 (0.43)

SAM, Survey of Autobiographical Memory; MLSQ, Motivated Learning Strategies Questionnaire; SBC, Study Behaviors Checklist; OSIQ, Object and Spatial Imagery Questionnaire.

The VVIQ significantly predicted scores on the ASSIST Strategic subscale, *β* = 0.056, 95% CI [0.017, 0.095], *F*(1, 405) = 7.671, *p* = 0.006, *p*_*adj*_ = 0.024, R^2^ = 0.019, 95% CI [0.003, 0.048], and the Deep subscale, *β* = 0.043, 95% CI [0.004, 0.082], *F*(1, 405) = 4.708, *p* = 0.031, *p*_*adj*_ 0.083 (not significant after FDR correction), R^2^ = 0.012, 95% CI [0.000, 0.036]. No significant relationship was found between VVIQ scores and the Surface subscale, *β* = −0.014, 95% CI [−0.058, 0.030], *F*(1, 405) = 0.472, *p* = 0.492, *p*_*adj*_ = 0.574, R^2^ = 0.001, 95% CI [0.000, 0.014]. For the teaching preferences extension of the ASSIST, VVIQ scores did not significantly predict preferences for either teaching approaches that Transmit Information, *β* = 0.016, 95% CI [−0.003, 0.035], *F*(1, 405) = 2.842, *p* = 0.093, *p*_*adj*_ = 0.149, R^2^ = 0.007, 95% CI [0.000, 0.025], or teaching approaches that Support Understanding, *β* = 0.019, 95% CI [−0.007, 0.045], *F*(1, 405) = 2.023, *p* = 0.156, *p*_*adj*_ = 0.208, R^2^ = 0.005, 95% CI [0.000, 0.021]. Additional analyses comparing hyperphantasic (scores 75–80) and aphantasic (scores 16–24) groups revealed no significant differences in deep, strategic, or surface learning approaches (*p* > 0.368).

The VVIQ demonstrated the strongest predictive relationships with the SAM subscales. VVIQ scores significantly predicted Autobigraphical Memory, *β* = 0.028, 95% CI [0.021, 0.035], *F*(1, 405) = 63.310, *p* < 0.001, *p*_*adj*_ < 0.001, R^2^ = 0.135, 95% CI [0.089, 0.186] (large effect), and Episodic Memory, *β* = 0.018, 95% CI [0.012, 0.024], *F*(1, 405) = 36.058, *p* < 0.001, *p*_*adj*_ < 0.001, R^2^ = 0.082, 95% CI [0.044, 0.125] (moderate effect). A weaker but still significant relationship was found with Semantic Memory, *β* = 0.007, 95% CI [0.001, 0.013], *F*(1, 405) = 5.597, *p* = 0.018, *p*_*adj*_ = 0.048, R^2^ = 0.014, 95% CI [0.001, 0.037] (small effect). VVIQ scores did not significantly predict Spatial Memory, *β* = 0.006, 95% CI [−0.001, 0.013], *F*(1, 405) = 2.716, *p* = 0.100, *p*_*adj*_ = 0.154, R^2^ = 0.007, 95% CI [0.000, 0.025]. Group comparison analyses revealed significant differences in both Episodic Memory, *F*(2, 433) = 9.98, *p* < 0.001, partial η^2^ = 0.044, 95% CI [0.014, 0.081] (small-to-medium effect), and Future Thinking, *F*(2, 438) = 18.04, *p* < 0.001, partial η^2^ = 0.076, 95% CI [0.035, 0.123]. For Episodic Memory, hyperphantasics (*M* = 3.96, SD = 0.75) scored significantly higher than aphantasics (*M* = 3.11, SD = 0.97, Cohen’s *d* = 0.98). Similarly, hyperphantasics (*M* = 4.19, SD = 0.71) demonstrated significantly higher scores on the Future Thinking subscale compared to aphantasics (*M* = 2.15, SD = 1.06, Cohen’s *d* = 2.21). No significant differences were found on the Semantic or Spatial subscales of the SAM (*p* > 0.108).

The VVIQ demonstrated limited predictive relationships with the MLSQ after FDR correction. VVIQ scores significantly predicted Self-Efficacy, *β* = 0.013, 95% CI [0.005, 0.021], *F*(1, 405) = 10.239, *p* = 0.001, *p*_*adj*_ = 0.008, R^2^ = 0.025, 95% CI [0.005, 0.053]. No significant relationships were found between VVIQ scores and Intrinsic Value, *β* = 0.007, 95% CI [−0.001, 0.015], *F*(1, 405) = 3.099, *p* = 0.079, *p*_*adj*_ = 0.133, R^2^ = 0.008, 95% CI [0.000, 0.026], or Test Anxiety, *β* = −0.001, 95% CI [−0.013, 0.011], *F*(1, 405) = 0.007, *p* = 0.933, *p*_*adj*_ = 0.933, R^2^ = 0.000, 95% CI [0.000, 0.009]. The group comparison analysis revealed a significant difference for Intrinsic Motivation, *F*(2, 438) = 5.64, *p* = 0.004, partial η^2^ = 0.025, 95% CI [0.003, 0.056]. *Post hoc* analyses showed that hyperphantasics (*M* = 6.15, SD = 0.72) demonstrated significantly higher Intrinsic Motivation compared to aphantasics (*M* = 5.53, SD = 0.90, Cohen’s *d* = 0.75). No significant differences were found in Self-Efficacy or Test Anxiety (*p* > 0.05). Due to an administration error during data collection, the Cognitive Strategy and Self-Regulation subscales contained missing data for 280 participants. Given the high proportion of missing cases and insufficient auxiliary variables required for reliable imputation procedures, these two subscales were excluded from all analyses.

Vividness of Visual Imagery Questionnaire scores significantly predicted three of the four SBC factors: Organization Behaviors, *β* = 0.015, 95% CI [0.009, 0.021], *F*(1, 405) = 21.526, *p* < 0.001, *p*_*adj*_ < 0.001, R^2^ = 0.051, 95% CI [0.021, 0.087]; Explanation Behaviors, *β* = 0.014, 95% CI [0.007, 0.021], *F*(1, 405) = 18.742, *p* < 0.001, p_FDR < 0.001, R^2^ = 0.044, 95% CI [0.016, 0.077]; and Testing Behaviors, *β* = 0.015, 95% CI [0.007, 0.023], *F*(1, 405) = 15.378, *p* < 0.001, *p*_*adj*_ = 0.001, R^2^ = 0.037, 95% CI [0.012, 0.067]. No significant relationship was found with Note Taking Behaviors, *β* = 0.006, 95% CI [−0.002, 0.014], *F*(1, 405) = 2.157, *p* = 0.143, *p*_*adj*_ = 0.191, R^2^ = 0.005, 95% CI [0.000, 0.021]. Significant group differences emerged in overall scores on the SBC, *F*(2, 424) = 6.04, *p* = 0.003, partial η^2^ = 0.028, 95% CI [0.004, 0.058]. *Post hoc* comparisons indicated that hyperphantasics (*M* = 3.83, SD = 0.69) scored significantly higher on the SBC than aphantasics (*M* = 3.18, SD = 0.53), Cohen’s *d* = 1.06. An examination of the individual factors revealed that hyperphantasics scored significantly higher on both the Testing Behaviors factor, *F*(2, 441) = 4.96, *p* = 0.007, partial η^2^ = 0.022, 95% CI [0.002, 0.049], and the Explanation Behaviors factor, *F*(2, 441) = 3.73, *p* = 0.025, partial η^2^ = 0.017, 95% CI [0.001, 0.041]. For the Note Taking Behaviors factor, Levene’s test indicated heterogeneity of variances (*p* = 0.027). Welch’s robust test of equality of means confirmed the non-significant difference between groups, *F*(2, 14.98) = 0.68, *p* = 0.522.

An analysis of scores on the Object and Spatial Imagery Questionnaire (OSIQ) revealed substantial group differences in object imagery, *F*(2, 434) = 34.21, *p* < 0.001, *p*_*adj*_ < 0.001, partial η^2^ = 0.136, 95% CI [0.078, 0.197]. *Post hoc* analyses showed that hyperphantasics (*M* = 4.19, SD = 0.43) scored significantly higher than aphantasics (*M* = 2.31, SD = 0.64, Cohen’s *d* = 3.41). Consistent with previous research, no significant differences were found in spatial imagery scores, *F*(2, 430) = 0.37, *p* = 0.693, *p*_*adj*_ = 0.731.

### Study 1 discussion

Study 1 addressed our first research question by investigating the relationship between visual imagery vividness and various learning and study strategies in college students. The results revealed significant patterns of findings that advance our understanding of cognitive differences across the visual imagery spectrum. Specifically, hyperphantasic participants demonstrated significantly more use of practice testing and example generation while studying, which are both strategies that likely leverage their enhanced ability to create and manipulate mental images. The higher intrinsic motivation experienced by hyperphantasic participants suggests they may find more inherent enjoyment in academic tasks, while the significant differences in object imagery, episodic memory, and future thinking ability align with theoretical frameworks suggesting that visual imagery serves as a cognitive scaffold for autobiographical and prospective memory processes, both of which are impaired in individuals with aphantasia (Beran et al., 2023; Dawes et al., 2022).

Our predictions regarding study approaches were partially supported. While we found significant relationships between VVIQ scores and strategic approaches, we found no significant differences in deep, strategic, or surface-level learning approaches when comparing aphantasic and hyperphantasic groups. This finding is particularly noteworthy given the theoretical complexity underlying our initial hypotheses. We had initially hypothesized that aphantasic students might rely more on surface-level approaches, but as we acknowledged, this prediction was theoretically problematic–surface approaches often emphasize visual features and rote memorization that may themselves depend on visual processing capabilities. The absence of differences across groups suggests that aphantasic students have developed sophisticated compensatory strategies that allow them to employ similar learning approaches to their peers, rather than being constrained to particular approach patterns.

The lack of differences in learning approaches across imagery ability levels may reflect the fact that by college age, students without mental imagery have already developed sophisticated unconscious compensatory strategies, placing them on par with their peers in terms of general study approaches. Additionally, our predominantly first-year participants may not have fully developed college-level study skills yet. Finally, our measures may not have been sensitive enough to capture the task-specific adaptations that aphantasic students employ.

Study 1 exclusively relied on self-reported quantitative measures, which limited its ability to explore the nuanced experiences and adaptive strategies of students with aphantasia. While we identified statistically significant relationships between VVIQ scores and learning outcomes, our standardized measures could not capture the lived experiences of aphantasic students, their perspectives on how this condition affects their academic pursuits, or the unique compensatory strategies they employ. By college age, individuals without visualization capabilities have likely developed unconscious adaptations to function in learning environments designed for those with typical imagery abilities. This adaptation process makes it challenging to assess the role of visualization in learning through traditional quantitative methods. As Larner et al. (2024) note, “the cognitive differences associated with aphantasia are often subtle and domain-specific, making them difficult to detect using general cognitive assessments.” To gain a more comprehensive understanding of the relationship between aphantasia and learning processes, Study 2 employs qualitative interviews with aphantasic students to explore their adaptive strategies and educational experiences in depth. This mixed-methods approach allows us to triangulate findings and develop a more nuanced understanding of how aphantasic students navigate higher education despite lacking visual mental imagery.

## Study 2

The quantitative findings from Study 1 provided valuable insights into the relationship between imagery ability and various cognitive and academic measures but left several important questions unanswered. While the results confirmed significant differences in certain memory domains and study behaviors, the unexpected similarities in learning approaches across the imagery spectrum suggest more complex cognitive adaptations at work than our quantitative measures could capture.

To address these limitations, Study 2 uses a qualitative methodology that focuses on the contextualized experiences of aphantasic students. This approach allows us to explore the specific mechanisms, thought processes, and compensatory strategies aphantasic students develop throughout their educational journeys. Qualitative interviews can reveal nuanced aspects of learning that standardized measures might miss, such as the evolution of study techniques over time, the broader implications of learning without mental imagery, and the nature of academic problem-solving in specific disciplines.

Additionally, while quantitative approaches excel at identifying patterns across groups, qualitative methods are particularly valuable for understanding phenomena where substantial individual variation exists. Because aphantasia affects approximately 2%–5% of the population (Zeman, 2024) and research on aphantasia is still in its infancy, an in-depth exploration of individual experiences can highlight both common adaptations and unique personal strategies. These findings can potentially inform more inclusive educational practices that accommodate diverse cognitive styles while offering insights into the remarkable flexibility of human cognition.

### Method

#### Participants

A total of 14 aphantasic students from a large Midwestern state university took part in this project in exchange for credit toward a class research requirement. Thirteen participants (93%) self-identified as female, and one participant (7%) self-identified as male. All participants were over the age of 18.

#### Materials

##### Vividness of visualization imagery questionnaire (VVIQ)

The VVIQ was given to students during prescreening to determine eligibility for inclusion in Study 2; the scale is described in detail in the Section “Materials” in Study 1. Students scoring less than 24 on the VVIQ were classified as aphantasic, following Zeman et al.’s (2020) recommendations. This threshold has been validated in recent research, with Beran et al. (2023) demonstrating that this cutoff yields prevalence rates consistent with established estimates (approximately 1.5%) and captures individuals who perform significantly differently on memory tasks compared to those with typical imagery.

### Procedure

During the first month of each semester, the Department of Psychology distributes a prescreening questionnaire to gather a variety of data on participants from the introductory psychology participant pool; the VVIQ was included as a measure of visual imagery. After the prescreening data were collected, total scores on the VVIQ were calculated and all students scoring less than 24 (*n* = 43) were contacted via email and invited to participate in a 1-h in-person interview to discuss experiences as a college student with aphantasia in exchange for credit toward their course research requirement. Individuals who did not respond to the initial email were sent one follow-up email several weeks later. Participants who responded to the solicitation email were scheduled for an in-person interview in a private office on campus. A total of 14 interviews were conducted; two members of the research team were present during every interview. To mitigate potential bias, interviewers were not acquainted with the students prior to the study. Additionally, all identifying information was removed from the transcripts prior to coding. All interviews were recorded.

#### Interview protocol

Interviews followed a loose structure that included three basic topic areas:

1. Academic Areas of Study. This topic area included questions about the student’s major and academic interests. Questions included, “What is your major/what major are you leaning toward?” “What are your favorite/least favorite subjects?” and “Are there subjects that are harder/easier to study for, and why?”
2. Memory and Retention. This topic area included questions about students’ experiences with memory, information retention, and study habits. Questions included, “Compared to others, do you feel like you struggle to remember certain types of information?” and “Are there certain topics that stick in your memory better than others?” Additionally, participants were briefly shown an object, and then asked to recall details about its appearance once it was out of sight. Following the student’s recollection of the object’s features, the object was brought back out and shown to the participant again.
3. Teaching and Learning Preferences. This topic area included questions about students’ preferred methods of learning and the types of teaching tools they find helpful. Questions included, “How many hours per week do you study?” “What kind of settings do you prefer for studying? (e.g., noisy, quiet, etc.),” “What do you think is your biggest difficulty regarding studying?” and “Are there common study techniques that work best/worst for you?” Students were also asked questions that directly related to visualization, such as, “If you are shown a diagram or a model in class, how do you commit it to memory?”

Follow-up questions were asked when participant responses required further clarification. After the interviews were complete, all recordings were transcribed by an undergraduate research assistant. Following the completion of the transcription process, interviews were coded using thematic analysis.

#### Analysis

The coding and analysis followed Braun and Clarke’s (2006) six-phase approach to thematic analysis. Phase 1 involved familiarizing ourselves with the data through repeated readings of the transcripts. In Phase 2, initial codes were generated across the entire dataset in a systematic fashion. The qualitative data analysis software Quirkos 2.5.3 (Quirkos, 2023) was used to support the organization and analysis of data. Initial coding categories included broad areas such as “memorizing information,” “use of media,” “studying behaviors,” and “academic major.” For example, discussions about techniques for remembering models and equations were coded under “memorizing information,” while discussions about interactions with various media forms, such as books, TV shows, and movies, were coded under “use of media.”

As the coding process progressed, we identified more specific patterns and developed subcategories to better define the categories created during Phase 2. For example, subcategories of “repetition,” “association,” “simplification,” and “verbalization” were added under the broader “studying behaviors” category. Data extracts were coded inclusively, retaining relevant surrounding context, and individual extracts were often coded into multiple categories to capture their full meaning. To ensure coding consistency, a second round of analysis was conducted by the primary coder, and two additional research team members independently coded portions of the transcripts to establish intercoder agreement.

In Phase 3, we searched for potential themes by collating codes into broader patterns of meaning. During Phase 4, we reviewed these potential themes to ensure they formed coherent patterns in relation to both the coded extracts (Level 1) and the entire dataset (Level 2). The research team collaboratively discussed the underlying meaning of each potential theme and identified conceptual overlap or distinction between them, developing a thematic map that evolved throughout the analysis.

Phase 5 involved defining and naming the themes, identifying the essence of each theme and determining how it contributed to understanding the data in relation to our research questions. Rather than focusing solely on categorical analyses, we emphasized the identification of patterns that appeared across multiple data items and how they interconnected to tell a coherent story about aphantasic students’ learning experiences. Finally, in Phase 6, we selected compelling extract examples and constructed an analytic narrative that moved beyond description to interpretation, connecting our findings to broader theoretical perspectives on learning without mental imagery.

### Results

Analysis of our interview data revealed four major themes that characterize how aphantasic college students approach learning. These themes represent distinct but interconnected compensatory mechanisms that aphantasic students have developed to succeed academically despite lacking visual mental imagery. Understanding how these students have adapted their cognitive processes to overcome limitations in visualization may provide valuable insights into alternative pathways for learning and information processing.

#### Theme 1: externalization as a compensatory strategy

Students with aphantasia rely heavily on external systems rather than internal visualization to organize and remember information. Participants consistently described needing to physically document information rather than trying to hold it in memory, often through extensive list-making and note organization systems. While list-making is common among many college students, aphantasic participants described an intense dependence on lists for basic daily functioning and organization. As one student explained:

I started with… a list of… what was happening in my classes,… so it’s like general stuff…then I had to make another list of my week, and then I had a next week one so that I could remember what I was doing next week. But then, that didn’t include the stuff that I should be doing right now, and so then I made a to-do right now list, but then, that got too busy, and so I made another list, and this is the stuff I need to do today.

This excerpt illustrates how participants develop elaborate systems of multiple lists to externally represent information they cannot visualize mentally. The progressive creation of more specific lists suggests an attempt to compensate for the inability to hold and manipulate schedule information through mental imagery, especially as their day grows increasingly busy.

The essential nature of list-making was emphasized across interviews, with participants describing lists as crucial to their basic functioning. One student stated plainly, “I probably would not do very well if I couldn’t make lists,” while another noted “I have to have everything very organized… I’m very list-oriented.” The severity of dependence on lists was particularly evident when participants considered scenarios without access to their lists, with one student explaining: “I’m off for the rest of the day. What am I- how am I supposed to remember what I’m gonna do?”

This reliance on external organizational aids appears closely connected to participants’ aphantasia, as illustrated by one student who explained that lists “help me a lot and keeps me a lot more organized ‘cause otherwise, I’m just kind of like going around through the day not knowing what’s happening or what’s next.” This suggests that in the absence of mental visualization abilities, lists serve as a crucial external scaffold for information that others might maintain through visual imagery.

#### Theme 2: verbalization and linguistic processing as a learning strategy

A second major theme that emerged from the interviews was participants’ strategic use of verbalization to compensate for their lack of visual imagery. Participants described employing both external (spoken) and internal (mental) verbalization to enhance their learning and memory. This verbalization appeared to serve multiple cognitive functions that might typically be supported by visual imagery in non-aphantasic individuals.

The fundamental nature of this linguistic processing strategy was clearly demonstrated during an object description task in the interviews. When briefly shown a container and then asked to describe it from memory, participants consistently defaulted to listing basic characteristics. One student recalled: “It was blue, it was long, like a cylinder. It had a handle on it and a white lid … There were ridges on the… top.” Notably, when the object was presented again, the same student reflected:

This is not what I imagined the ridges to be like in my head. Again, I was like “ridges” and I couldn’t remember what the ridges were like, but I remember thinking in my head the word “ridges,” so I know that there were [ridges].

This metacognitive insight reveals how aphantasic individuals may encode visual information primarily through verbal labels rather than mental images. This linguistic approach extends to deliberate study strategies. External verbalization was frequently described as a key learning tool. As another participant explained, they would:

Say the different people we had to memorize out loud, and then I would tell him the story about them… That’s the way I can remember studying recently, is like saying it out loud, repeating what I remember.

This systematic approach to verbal rehearsal was echoed by another student, who described combining verbalization with writing:

It just helps me remember it better because if I’m writing it down, I also like saying it out loud… I have to… systematically repeat it… out loud so that… whenever I get the piece of paper I write it down as fast as I can so that I’ll have it there.

Beyond individual study, participants also emphasized the importance of verbal discussion for processing academic material. As one student noted, they “… can’t read the textbook and it makes sense to me without… being able to talk it through with somebody.”

Internal verbalization, or inner speech, emerged as another key strategy. Participants described using their “internal dialogue” to support both comprehension and memory. This was particularly evident in one participant’s description of working with challenging material: “When I am struggling to understand something that I’m reading, if I read it out loud and hear myself reading it, it suddenly makes more sense.”

These accounts suggest that aphantasic students may rely more heavily on verbal processing pathways to compensate for their inability to create and manipulate visual mental images. The systematic nature of their verbal strategies - combining inner speech, spoken rehearsal, and discussion with others - appears to provide an alternative route to learning and memory that bypasses the need for visual imagery.

#### Theme 3: using familiar references to anchor narrative information

A third significant theme that emerged from the interviews was participants’ systematic strategy of mapping new information onto existing knowledge structures to aid comprehension and memory. This appeared to be a key compensatory mechanism for processing narrative information in the absence of visual imagery. The core of this strategy involves using familiar reference points as cognitive anchors. As one participant explained: “Whenever I see a character in the book … I base them off of someone I’ve seen before… or… someone I know… like a point of reference.” This process of anchoring new information to existing knowledge serves as a foundational strategy that supports further understanding. As the same participant elaborated, they “… would automatically pull someone from something else, and just use them as… a template.” This suggests a systematic approach where unfamiliar elements are processed by connecting them to established mental representations.

This strategy appeared consistently across participants, with multiple students describing similar approaches. One student noted they “relate them to, like, real people. ‘Cause it’s easier for me to understand. Or like, movie, like famous people.” Another explicitly connected this strategy to their aphantasia, explaining that they “relate [the characters] to people that I know, or that I have seen because I can’t actually… visualize them.” One gave the example: “When they said grandma in Little Red Riding Hood I was like “okay an old white lady”… I was like, she has like the stereotypical, like gray curly hair and glasses.” This theme reveals how aphantasic individuals may develop alternative pathways for processing novel information that would typically be supported by visual imagery. Rather than creating new mental images to represent unfamiliar concepts, they appear to build understanding through a network of connections to existing knowledge. This systematic linking of new to known information suggests a sophisticated cognitive adaptation that enables comprehension and memory without relying on visual imagery.

#### Theme 4: multi-modal strategies for navigating visual-heavy academic content

Another prominent theme emerging from the interviews was participants’ need to develop alternative approaches to learning when faced with traditionally visualization-dependent academic content. Rather than being able to create and manipulate mental images, participants described relying heavily on external representations and hands-on experiences to process information. The challenge of working with purely textual descriptions was highlighted by one participant’s frustration having to “deal with visualiz[ing] what’s going on” in exam questions: “And it’s just like, there’s this question and it’s like “Well, how is this supposed to play out?” when like it’s just words, like I, like I don’t see anything else playing out in the question.” This excerpt illustrates a fundamental difficulty faced by aphantasic students - the inability to automatically generate mental representations from written descriptions, which many traditional teaching methods assume students can do.

The importance of external visual aids emerged strongly in participants’ accounts. One student emphasized their need to physically “see the picture” to understand concepts such as those presented in molecular chemistry, suggesting that aphantasic individuals may rely more heavily on concrete external representations than their peers:

All of [the students] would, like, “Oh, I get that,” and then, but I would have to, like, see the picture in order to relate it to the shape, like, what it is called … when it came to like … tetrahedral and, like, the 3-D shapes, it was hard for me to, like, understand it. All of my classmates would, like, get it … I didn’t understand how ‘cause I couldn’t see it.

Hands-on learning emerged as particularly valuable, as illustrated by one participant’s experience with anatomical dissection. They described how having the physical object “in front of [them]” was “a lot cooler” and more effective than studying from “a slide show.” This suggests that direct physical interaction with learning materials may provide an alternative pathway to understanding that bypasses the need for mental visualization.

Compensatory strategies extended to how information was organized and stored for later retrieval. As one participant described: “I write out all my steps or I miss things because I just go off memory … Because I can’t see it.” Another noted: “I’d rather have a chart that boils down to words… Like I kind of just skip past pictures in books cause I’m like I’m not gonna remember.” This preference for breaking down visual information into verbal or written components that could be systematically processed and reviewed emerged as a key compensatory strategy.

These accounts reveal how aphantasic students actively develop alternative learning strategies when traditional visualization-dependent approaches are insufficient. Their experiences suggest that successful learning for aphantasic individuals often involves finding concrete external substitutes for the internal visualization processes that many educational methods take for granted.

### Study 2 discussion

The four major themes identified in Study 2 reveal an integrated system of compensatory mechanisms that aphantasic students use to succeed academically. Analysis of these themes reveals a fundamental pattern: aphantasic students systematically externalize cognitive processes that are typically internalized through visual imagery in other students. This externalization manifests across multiple interconnected domains. In terms of information organization and retrieval, list-making and verbalization work together as complementary strategies. While lists provide external structure, verbalization offers a way to process and reinforce that structure, creating a robust system for managing both academic content and daily tasks. For content processing and understanding, verbalization intersects with the use of familiar references to create a dual-processing approach. Students use verbal labels to tag and categorize information while simultaneously anchoring new concepts to existing knowledge, creating a network of interconnected information that compensates for the inability to create mental images. These strategies are further enhanced by multi-modal approaches to learning, where physical representations and hands-on experiences provide concrete anchors for verbal descriptions, and lists often incorporate multiple modalities including written words, diagrams, and physical organization.

Understanding these intersecting themes not only illuminates how aphantasic students succeed academically but also provides valuable insights for creating more inclusive educational environments. The sophisticated compensatory strategies developed by aphantasic students demonstrate the brain’s remarkable adaptability and suggest approaches that could benefit all students, regardless of their visualization abilities. By recognizing and supporting these alternative learning pathways, educators can create more effective and inclusive learning environments that accommodate diverse cognitive profiles.

## General discussion

This two-part mixed-methods study explored how college students with aphantasia–the inability to voluntarily generate visual mental imagery–navigate academic environments. Our research revealed sophisticated compensatory mechanisms that allow aphantasic students to succeed academically despite lacking visual mental imagery, a cognitive ability typically considered fundamental to learning processes.

Study 1 employed quantitative measures to examine relationships between visual imagery ability and academic variables across the imagery spectrum. Aphantasic students reported significant differences in episodic memory, future thinking, and certain study behaviors compared to those with hyperphantasia. Notably, hyperphantasic students were more likely to use practice testing and explanation-based study strategies, which correspond with evidence-based practices for effective learning (Guarnera et al., 2019; Jenkins, 2009; Leopold and Mayer, 2015). However, contrary to our hypotheses, we found no significant differences in deep, strategic, or surface learning approaches across the imagery spectrum, suggesting that by college age, aphantasic students have developed effective compensatory mechanisms that place their overall learning approaches on par with peers.

Study 2 provided rich qualitative insights into these compensatory mechanisms through in-depth interviews with aphantasic college students. Four major themes emerged: (1) Externalization as a compensatory strategy: aphantasic students rely heavily on external organizational systems like extensive list-making rather than internal visualization; (2) Verbalization and linguistic processing: they systematically employ verbal strategies, both internal and external, to encode and retrieve information; (3) Using familiar references to anchor narrative information: they map new concepts onto existing knowledge structures to aid comprehension; and (4) Multi-modal strategies for navigating visual-heavy content: they use external representations and hands-on experiences to process information that is typically dependent on mental imagery.

### Theoretical implications

Collectively, these findings reveal an integrated system of compensatory mechanisms that aphantasic students employ across academic contexts. The fundamental pattern suggests that aphantasic students systematically externalize cognitive processes that are typically internalized through visual imagery in other students, creating alternative pathways for learning and information processing. Our findings align with recent theoretical frameworks proposing that imagery vividness reflects part of an “inwardly focused” cognitive style that encompasses interoceptive attention, emotional awareness, and related cognitive processes (Kvamme et al., 2024). The compensatory strategies we identified–externalization through list-making, systematic verbalization, anchoring to familiar references, and multi-modal approaches–can be understood as adaptations to a reduced inward focus. Aphantasic students’ reliance on lists, verbal labels, and concrete external supports represents the outward scaffolding of processes that, in typical imagers, are internalized through an introspective, imagery-based focus. This external-versus-internal distinction provides a unifying framework for understanding the diverse compensatory mechanisms aphantasic students employ across academic contexts.

Additionally, our findings have important implications for theoretical understanding of the relationship between mental imagery and learning. From a strict Dual Coding Theory perspective (Paivio, 1991), aphantasic students should be substantially disadvantaged, limited to verbal encoding without access to the imagery system that provides redundant memory traces and retrieval pathways. However, our results paint a more complex picture that challenges simplistic applications of this framework.

The absence of differences in overall learning approaches (deep, strategic, and surface) between aphantasic and non-aphantasic students in Study 1 suggests that visual imagery, while potentially beneficial, is not necessary for employing the full range of study strategies. This finding aligns more closely with multi-component working memory models (Baddeley, 2000) and embodied cognition perspectives (Barsalou, 2008), which propose that multiple cognitive systems can compensate for one another in supporting learning and memory.

This distributed approach bears some resemblance to Dual Coding Theory in that it involves multiple representational formats–but these formats are not the verbal and imagery systems Paivio proposed. Instead, aphantasic students appear to create a compensatory system that leverages external representations, verbal encoding, spatial processing (which remains intact in aphantasia; Dawes et al., 2020), and other sensory modalities to achieve learning outcomes comparable to their peers. This suggests that the “dual” in Dual Coding may be less about specific verbal and visual systems, and more about the general principle that multiple, diverse representations enhance learning–regardless of whether those representations are visual.

Our findings also support and extend theories of cognitive flexibility and neural plasticity. The fact that aphantasic students develop these compensatory strategies largely unconsciously and achieve academic outcomes on par with their peers suggests that the cognitive systems supporting learning are more flexible and adaptive than traditional theories have recognized. This aligns with recent work emphasizing the brain’s capacity for functional reorganization when typical pathways are unavailable (Milton et al., 2021; Keogh et al., 2021).

Importantly, our findings challenge the assumption that visual imagery is necessary for deep learning approaches. Deep learning is typically characterized by creating meaningful connections to existing knowledge structures, which is a process often assumed to depend on visual-conceptual anchoring. However, the absence of differences in deep learning approaches across imagery levels suggests that these meaningful connections can be developed through multiple pathways: verbal elaboration, associative networks, spatial relationships, or embodied understanding. This finding has important implications for instructional design, suggesting that teaching methods need not assume or require visual imagery to promote deep learning.

Our findings demonstrate that visual imagery, while advantageous for some, is not necessary for successful academic learning. Multiple cognitive pathways–including external representations, verbal encoding, spatial processing, and embodied understanding–can support the full range of learning approaches. This cognitive flexibility has important implications for both educational theory and practice, suggesting that effective instruction should accommodate multiple pathways to learning rather than privileging visualization-dependent approaches.

### Empirical implications

Our observations regarding aphantasic students’ difficulties with episodic memory and future thinking align with research by Dawes et al. (2022), who found impairments in autobiographical and prospective memory processes in individuals with aphantasia. However, our qualitative findings extend this work by revealing the specific strategies aphantasic students develop to overcome these challenges, such as detailed list-making and verbal encoding of experiences.

Keogh et al. (2021) observed that aphantasics perform equally well on visual working memory tasks even though they use different neural mechanisms. Our study extends this finding by demonstrating that aphantasic students develop alternative cognitive strategies that allow them to succeed academically across diverse learning contexts. The compensatory strategies we identified–particularly the reliance on verbalization, external representations, and associative networks–provide potential explanations for why aphantasics show no deficits in standardized measures of cognitive ability despite lacking visual imagery (Dawes et al., 2020). Similarly, while Cui et al. (2007) demonstrated that aphantasics excel at describing spatial relationships despite differences in visual cortex activity, our study illuminates how this might occur through alternative cognitive pathways, including the systematic use of verbalization and external references.

### Limitations

Investigating the experiences of individuals with aphantasia is inherently challenging, especially given the established relationship between aphantasia and autobiographical memory difficulties. Many participants reported a lack of clear insight into their earlier educational experiences, which affected their ability to compare current study approaches with those used prior to college. Additionally, interview participants often needed time to process the concept of aphantasia itself before reflecting on how their educational experiences might be understood through the lens of limited visual mental imagery. Their inexperience with aphantasia as a concept likely hindered their ability to think deeply about its influence on their lives.

The measurement of aphantasia itself presents another challenge. We relied on self-report measures (specifically, the VVIQ) to categorize students as aphantasics. However, without incorporating objective physiological measures [such as those used by Kay et al. (2021) or Keogh and Pearson (2018)], we are unable to fully validate participants’ subjective ratings of their internal experiences. While common, this reliance on self-report introduces potential measurement error that could affect our findings. Additionally, while we followed established guidelines for VVIQ-based classification (Zeman et al., 2020), the field continues to evolve in its understanding of how to optimally define and measure imagery extremes. The distinction between extreme and moderate aphantasia, while conceptually meaningful, may have practical implications for research findings that warrant further investigation.

Similar to Dawes et al. (2022), we acknowledge the potential for self-report bias in our methodology. Participants who are aware of their aphantasia status might underestimate their cognitive abilities due to heightened awareness of their visualization limitations. Conversely, once introduced to the concept of aphantasia, participants might retrospectively interpret their learning experiences through this lens, potentially reporting strategies they believe align with the condition rather than those they actually employ. This could introduce confirmation bias into our qualitative findings.

Additionally, the composition of our participant sample presented several limitations. The predominance of first-year college students may have affected our results, as these participants likely had not fully developed academic study habits or the metacognitive awareness typical of more experienced college students. Their limited exposure to college-level academic demands might restrict the range of compensatory strategies they have needed to develop. This was evident in at least one case where a participant reported never needing to study, potentially limiting the insights available from their experience.

The sample also lacked diversity in institutional context and demographic representation. The limited disciplinary diversity makes it difficult to capture potential variations in compensatory strategies across different academic fields. For example, students in highly visual disciplines (such as art, architecture, or certain sciences) might develop different adaptations compared to those in text-heavy fields. These sampling limitations potentially restrict the generalizability of our findings to the broader population of students with aphantasia.

The small sample size (*n* = 14) and significant gender bias (93% female) in Study 2 presents multiple concerns for the validity and generalizability of our findings. First, the small sample size limits the range of compensatory strategies we may have captured, as different aphantasic students may develop varied approaches based on their academic disciplines, learning histories, and individual differences. Second, and perhaps more importantly, the gender bias introduces the possibility that the compensatory strategies we identified may be gender-specific rather than characteristic of aphantasic learners generally. Male and female students may develop different compensatory mechanisms for aphantasia based on socialized learning preferences, cognitive processing differences, or varying levels of comfort with different study strategies. For example, our findings regarding extensive verbalization and collaborative learning might reflect gendered learning preferences rather than aphantasia-specific adaptations. Future research should prioritize recruiting gender-balanced samples and larger sample sizes to determine whether the compensatory strategies we identified are truly characteristic of aphantasic learners or reflect gender-specific adaptations to the absence of mental imagery.

Finally, it is difficult to determine whether the strategies we observed were specifically developed to compensate for aphantasia or are general learning approaches that would have developed regardless of imagery ability. Without longitudinal studies tracking the development of these strategies from early childhood through higher education, we are unable to definitively attribute them to aphantasia-related adaptation. A more complex research design would be needed to determine whether the observed compensatory mechanisms are unique to aphantasia or represent more general cognitive adaptations.

### Suggested classroom applications

Our findings suggest several approaches for enhancing instruction to better support aphantasic learners. For example, instructors may consider creating content presentations that provide clear organizational frameworks through outlines, concept maps, and structured information, as aphantasic students rely heavily on external organization of information. Incorporating explicit verbal descriptions along with visual materials is crucial, as these students benefit from detailed linguistic explanations that capture essential features and relationships that others might grasp visually. Instructors should also allow time for students to create their own personalized external representations of complex information, as these representations may not develop as naturally in aphantasic students.

Aphantasic learners will also likely benefit from having content presented through multiple channels–verbal, written, and tactile–rather than relying primarily on visualization exercises. The use of hands-on activities and physical models provides concrete representations that aphantasic students can engage with directly instead of being asked to mentally visualize concepts. Regular opportunities for verbal discussion and explanation allow these students to process information through linguistic pathways, strengthening their understanding through verbalization rather than visualization.

When possible, assessment methods should offer flexibility, providing multiple ways to demonstrate knowledge that does not rely exclusively on visual memory or visualization skills. Including options that emphasize verbal processing allows aphantasic students to leverage their strengths in linguistic encoding and retrieval. When appropriate, permitting the use of external organizational tools during assessments acknowledges these students’ reliance on external memory aids rather than internal visualization.

It should be noted that many of these suggestions are universally beneficial – that is, their recommendation as “best practices” is not exclusive to aphantasic students. However, aphantasic students may see a proportionally greater increase in learning as a result of these changes because they are unable to substitute the use of visualization techniques enjoyed by others. These recommendations acknowledge the sophisticated compensatory strategies that aphantasic students naturally develop while creating an educational environment that supports diverse cognitive profiles, ultimately benefiting all students regardless of their visualization abilities.

## Conclusion

Although research on aphantasia is still in its infancy, our two-part mixed-methods study provides valuable insights into the sophisticated compensatory mechanisms that students with aphantasia develop to effectively navigate academic environments. Our findings revealed significant differences in episodic memory, future thinking, and specific study behaviors between aphantasic and hyperphantasic students. Hyperphantasic students demonstrated greater use of practice testing and example generation while studying, strategies that likely leverage their enhanced visual imagery capabilities.

The compensatory strategies employed by aphantasic students consistently involve externalizing cognitive processes that others typically internalize through visualization. These include structured organizational systems like extensive list-making, systematic verbal processing for encoding and retrieving information, mapping new concepts onto familiar references, and using multi-modal approaches to comprehend visual-heavy academic content. Despite lacking visual mental imagery–a cognitive ability traditionally considered fundamental to learning–these unconscious compensatory mechanisms appear to enable aphantasic students to perform at the same level as their peers academically. The similar use of deep, strategic, and surface learning approaches across imagery groups, combined with the sophisticated compensatory strategies revealed in our qualitative data, suggests that multiple cognitive pathways can lead to successful academic outcomes.

These findings have important implications for educational practice. Instructors can better support diverse cognitive profiles by providing clear organizational frameworks for content, incorporating explicit verbal descriptions alongside visual materials, allowing for personalized external representations, and encouraging verbalization through discussion and collaboration. With approximately 2%–5% of the population experiencing aphantasia, recognizing these different learning approaches is essential for developing more inclusive and effective educational environments.

Ultimately, this research contributes to theoretical debates about the role of mental imagery in cognition by demonstrating that visual imagery, while potentially facilitative, is not necessary for successful academic learning. Our findings challenge strict interpretations of Dual Coding Theory and support more flexible, multi-component models of cognition that emphasize the brain’s capacity to achieve similar functional outcomes through diverse cognitive pathways. By understanding and supporting these diverse cognitive profiles, educators can create learning environments that benefit all students, regardless of their visualization abilities, while advancing our theoretical understanding of the multiple routes to successful learning.

## Data Availability

The raw data supporting the conclusions of this article will be made available by the authors, without undue reservation.
